# Cadmium‐tolerant bacteria: current trends and applications in agriculture

**DOI:** 10.1111/lam.13594

**Published:** 2021-11-13

**Authors:** D. Bravo, O. Braissant

**Affiliations:** ^1^ Laboratory of Soil Microbiology & Calorimetry Corporación Colombiana de Investigación Agropecuaria AGROSAVIA Mosquera Colombia; ^2^ Department of Biomedical Engineering Faculty of Medicine University of Basel Allschwill Switzerland

**Keywords:** agriculture, bioremediation, environmental health, food safety, pollutants

## Abstract

Cadmium (Cd) is considered a toxic heavy metal; nevertheless, its toxicity fluctuates for different organisms. Cadmium‐tolerant bacteria (CdtB) are diverse and non‐phylogenetically related. Because of their ecological importance these bacteria become particularly relevant when pollution occurs and where human health is impacted. The aim of this review is to show the significance, culturable diversity, metabolic detoxification mechanisms of CdtB and their current uses in several bioremediation processes applied to agricultural soils. Further discussion addressed the technological devices and the possible advantages of genetically modified CdtB for diagnostic purposes in the future.

## Graphical abstract

**Significance and Impact of the Study:** Crop production is adversely affected by the presence of heavy metals in soils dedicated to agriculture. These metals ultimately accumulate in seeds or edible parts and enter the human food chain. The application of cadmium (Cd)‐tolerant bacteria capacities for Cd immobilization and sequestration, in combination with soil management and implementation of other agronomic practices, may reduce the Cd levels in farmlands.

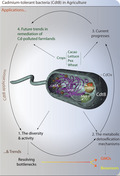

## Introduction

Cadmium (Cd) became an issue in human nutrition and health with the demonstration, that the mobility of this toxic heavy metal could allow it to follow a path from agricultural soils to end‐products in the human food chain. For instance, in the production of basic foods such as rice, lettuce or potatoes, Cd can be accumulated in the plant tissue at concentrations exceeding the levels that pose risks for human health (Chaney *et al*. 1999). Levels of 0·10 mg kg^−1^ are considered the allowable maximum value of Cd in food crops (Commission 2007; European Food Safety Authority 2009; FAO/WHO 2018; Hou *et al*. 2020). However, recent studies have shown that Cd is also an issue in raw materials such as cacao, from which chocolate is produced (Shahid *et al*. 2017; Anyimah‐Ackah *et al*. 2019). This issue has become of great importance for cacao‐producing countries in Central and South America in the last years (Chavez *et al*. 2015; Arévalo‐Gardini *et al*. 2017; Bravo *et al*. 2018; Gramlich *et al*. 2018). Interestingly, despite its cosmopolitan distribution, most of this metal content is not absorbed by humans because an important proportion of environmental Cd remains in unavailable forms in the soils (i.e. Greenockite (CdS), Otavite (CdCO_3_) or less frequent Monteponite (CdO) Traina 1999). This minerals cannot be absorbed by plants through their root systems (Welch and Norvell 1999) to enter the human food chain. Table 1 shows the most common natural Cd solid forms of interest found in soils (Traina 1999).

**Table 1:** Natural Cd solid forms occurring in soils. Adapted from Traina (1999) and Cook (2001)

Name	Molecular formula	Reaction	Equilibrium constant *K* ^θ^
Cadmoselite	CdSe	CdSe + 1·5 O_2_(_g_)↔Cd^2+^ + SeO_3_^2−^	53·60
Cadmium selenite	CdSeO_3_	CdSeO_3_↔Cd^2+^ + SeO_3_^2−^	−8·80
Greenockite/Hawleyite	CdS	CdS↔Cd^2+^ + S^2−^	−27·07
Cadmium silicate	CdSiO_3_(c)	CdSiO_3_ + 2H^+^ + H_2_O↔Cd^2+^ + H_4_SiO_4_^0^	7·63
Cadmium sulphate	CdSO_4_	CdSO_4_↔Cd^2+^ + SO_4_^2−^	−0·04
Dihydride Cd sulphate	CdSO_4_:2·66H_2_O	CdSO_4_:2·66 H_2_O ↔ Cd^2+^ + SO_4_^2−^ + 2·66 H_2_O	−1·80
Monteponite	CdO	CdO + 2H^+^↔Cd^2+^ + H_2_O	−13·64
Otavite	CdCO_3_	CdCO_3_ + 2H^+^↔Cd^2+^ + CO_2_(g) + H_2_O	13·65
Cadmium hydroxide	β‐Cd(OH)_2_(c)	β‐Cd(OH)_2_ + 2H^+^↔Cd^2+^ + 2H_2_O	15·14
Spinel	CdCr_2_O_4_(c)	CdCr_2_O_4_ + 8H^+^↔Cd^2+^ + 2Cr^3+^ + 4H_2_O	15·00
Cadmium phosphate	Cd_3_(PO_4_)_2_(c)	Cd_3_(PO_4_)_2_ + 4H^+^↔3Cd^2+^ + 2H_2_PO_4_	1·00

**Figure 1:**
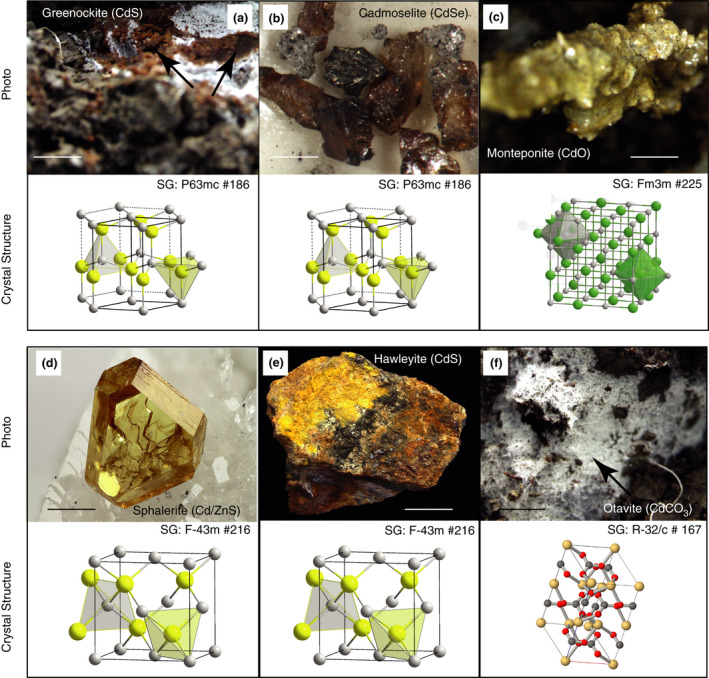
The main Cd solid forms with low solubilization that remain unavailable to plant uptake (photo) and their crystal structure, where a and b are isotypes of Wurtzite and c is an isotype of Halite. (a) Greenockite (CdS), (b) Cadmoselite (CdSe), (c) Monteponite (CdO), (d) Sphalerite (Cd/ZnS), (e) Hawleyite (CdS) and (f) Otavite (CdCO_3_). Different Cd solid forms have identical crystal structure because the stoichiometry of the Cd‐like compounds, high‐temperature–pressure oxide melting processes and the edaphoclimatic conditions developed in several farmland soils. Photos b (from Łukasz Kruszewski), d (Chinellato Matteo) and e (from J.F. Carpentier) were downloaded from Mindat.org with a magnification bar of 1 cm. Photos a, c and f were from Dr. Daniel Bravo, taken in subsoil of cacao‐growing farms in Colombia, with a magnification bar of 0·5 cm.

In other crops, such as lettuce and spinach, the pH and the Zn/Cd ratio influence in Cd availability for uptake and plant accumulation (McKenna *et al*. 1993; Tang *et al*. 2020). Figure 1 shows the main Cd solid forms that remains unavailable for plant root systems.

In farmland soils, the Cd content is due to geogenic conditions, however, maybe also due to human activities related to bad‐quality manures, amendments and fertilizer abuse (Gil *et al*. 2021), as well as to mining, oil pipelines, smelting, electroplating, irrigation using wastewater or Cd‐contaminated efflux water (Bolan *et al*. 2014).

Regardless of the point of entry, the first direct and indirect impacts of the Cd content in soils are felt by soil microorganisms, which come in contact with the Cd forms present in soil solutions or adsorbed as solid‐state phases (Brookes 1995). Cadmium‐tolerant bacteria (CdtB) have evolved, and these have become crucial for bioremediation purposes in agricultural soils that are Cd enriched (≥0·9 mg kg^−1^ of soluble Cd) or contaminated (≥1 mg kg^−1^ of soluble Cd) (Bravo *et al*. 2018). In farmlands, the maximum permissible Cd concentration in soils depend on the type of crop and health issues in the value food chain. It can vary in rice and wheat, from 0·01 to 0·4 and 0·8 mg kg^−1^ of Cd in cacao crop (Bravo *et al*. 2021). At this point, it is important that concepts such as the geoaccumulation index are defined. According to previous studies including polluted farmlands reviewed nationwide (Wang *et al*. 2015), the geoaccumulation index (*I*_geo_) was introduced by Müller (1969) and refers to the assessment of the degree of enrichment under anthropogenic pollution, the geochemical background values and the effect of natural diagenesis. The *I*_geo_ index take into consideration the Cd concentration measured in topsoil (mainly rhizospheric soil) and the geochemical background values of Cd found at the same sampling points. The *I*_geo_ index includes seven classes of soil quality where the highest class represents a 100‐fold enrichment compared to the background values of contamination. Nonetheless, despite the importance of the index, the way through which the transfer of Cd occurs from the soil to the food chain in farmlands remains unclear. Thus, the geomicrobiology of the soil assessed here becomes relevant to understand the flux of Cd in a specific crop.

Although studies of CdtB were initiated in the 1970s (Babich and Stotzky 1977), agricultural research began later using bacterial isolates obtained from wheat (Woolhouse 1983) and rice (Babich and Stotzky 1985). The increase in regulations in the past years focusing on food safety (Gallego *et al*. 2012) has resulted in the development of bioremediation approaches in the production of commodity crops such as cacao (Bravo *et al*. 2018).

Both, mobilizing and immobilizing of Cd are important in bioremediation. Mobilizing activity can be used to enhance the removal of Cd through plant uptake and soil washing, whereas immobilizing amendments will reduce the transfer of Cd into food chain via plant uptake and reduce leaching into groundwater (Bolan *et al*. 2014). The choice to ‘mobilize’ or to ‘immobilize’ agricultural soil Cd content will depend on the crop system, the feasibility of bioremediation to be selected and the biogeochemical properties of the surrounding system.

### Cd resistance or Cd tolerance—is there any difference?

The terms such as tolerance and resistance are used differently in several scientific fields, i.e. environmental and biomedical fields.

For instance, in medical field, the terms are used in opposite fashion to the environmental fields, and the amounts vary considerably, such as in clinical microbiology, where 20 mg kg^−1^ Cd is considered a small amount of the metal to produce synthetic media such as the CFAT and GMC media (Atlas 2010), to be used in dental plaque analysis (see Table 2).

**Table 2:** Synthetic media used for study culturable populations of CdtB in both environmental and medical fields

Name	Cd reagent	Conc. per litre	Composition per litre	Use	References
CFAT medium	CdSO_4_	0·13 g	Pancreatic digest of casein 17 g, agar 15 g, glucose 7·5 g, NaCl 5 g, papaic digest of soya bean meal 3 g, K_2_HPO_4_ 2·5 g, NaF 0·8 g, CdSO_4_ 0·13 g, K_2_TeO_3_ 2·5 mg, neutral acriflavine 1·2 mg, basic Fuchsin 0·25 mg, sheep blood, defibrinated 50 ml	Clinical: dental plaque	Atlas (2010)
GMC medium	CdSO_4_·8H_2_O	0·02 g	Solution 1 950 ml: gelatine, agar, pancreatic digest casein, NaCl, d‐mannitol, KNO_3_, sodium acetate, sodium succinate, yeast extract, CdSO_4_·8H_2_O, metronidazole, sodium lactate (60% solution) Solution 2 50 ml: Na_2_HPO_4_, l‐cysteine HCl·H_2_O, Na_2_CO_3_·H_2_O, sucrose, dithiothreitol, menadione solution	Clinical: dental plaque	Atlas (2010)
Aleksandrov	CdSO_4_	1 g	Agar 20 g, CaCO_3_ 0·1 g, Ca_3_PO_4_ 2 g, CdSO_4_ 1 g, FeCl_3_ 0·006 g, feldspars 4 g, glucose 5 g, MgSO_4_·7H_2_O 0·5 g	Environmental: geomicrobiology	Aleksandrov *et al*. (1967); Parmar and Sindhu (2013)
Schlegel	CdNO_3_	1 g	Na_2_HPO_4_·12H_2_O 9 g, KH_2_PO_4_ 1·5 g, NH_4_Cl 1 g, MgSO_4_·7H_2_O o·2 g, FeNH_4_‐citrate 1·2 mg, CaCl_2_ 20 mg, Hoagland‐solution 2 ml, NaHCO_3_ 0·5 g, CdNO_3_ 1 g	Environmental: biogeochemistry	Schlegel *et al*. (1961)
Mergeay	CdCl_2_·5H_2_O	4 g	Tris HCl 50 mmol l^−1^ 6·06 g, NaCl 80 mmol l^−1^ 4·68 g, KCl 20 mmol l^−1^ 1·49 g, NH_4_Cl 20 mmol l^−1^ 1·07 g, Na_2_SO_4_ 3 mmol l^−1^ 0·43 g, MgCl_2_ 6H_2_O 1 mmol l^−1^ 0·20 g CdCl_2_·5H_2_O 0·8 mmol l^−1^ 4 g	Environmental: heavy metals study	Mergeay (1995)

The concept of resistance for the geomicrobiological field refers only to survival mechanisms and does not include the capacity for reaching biomass production. Resistant microbes have altered their genetics and/or their physiology in well researched ways to become less susceptible to an antimicrobial agent (Chapman *et al*. 1998). By contrast, ‘tolerance’ has been neither defined nor studied in a systematic manner.

Tolerance is used to describe a situation in which a formerly effective system (or ecosystem) no longer controls microbial growth. Implicit in this definition are two concepts. One is that something has changed in the surroundings and the other is that tolerance development has several potential causes. Therefore, the concept of tolerance includes the capacity for reaching biomass production by bacterial populations once a pollution event has occurred in the environment.

In this review, we will refer to the tolerance term, that bacteria use the available Cd content in crop systems as a source of energy (Beveridge 1989; Aryal 2020) to activate various mechanisms. One of these mechanisms is the efflux pump, where is actively regulated the cellular oxidative stress and the Cd accumulation process (Wang *et al*. 2020).

Moreover, tolerance is a response of individual cells to disturbance, which has consequences on the stability of the total community and is related to the activation of protective or adaptative mechanisms for surviving.

Cadmium‐tolerant bacteria form a heterogeneous group that is present in soils, water and air (Dabir *et al*. 2019). Several terms such as resistant, tolerant or immobilizer‐like are applied to this group (Rojjanateeranaj *et al*. 2017; Shan *et al*. 2019; Yasmeen *et al*. 2019). All these terms refer the concept of bacteria surviving in an environment enriched or polluted with heavy metals, such as Cd (Lal *et al*. 2019). The first studies on Cd tolerance by microorganisms were conducted in 1920s (Baas‐Becking and Parks 1927) and later experiments were carried out with metal salts to assess microbiological behaviour and growth (Colley 1931). Further research was aimed at understanding the ecological importance of bacteria that are able to grow in a medium rich in soluble Cd (Winslow and Haywood 1931; Perlman 1945). Some specific media were designed to assess minerals, including heavy metals (Schlegel *et al*. 1961). Then, in the mid‐1990s, a study showed the use of a specific medium to target the adaptations of this bacterial functional group (Mergeay 1995). Table 2 shows some referential synthetic media developed to study culturable CdtB.

However, studies on understanding of Cd uptake by microorganisms are still scarces due to the limited availability of appropriate culture media. Indeed, no new synthetic media have been introduced for heavy metal‐tolerant bacteria since the decade of 1990s last century. Some studies have shown that metals influence bacterial populations, affecting their morphology and metabolic activity (Pal *et al*. 2005; Siripornadulsil and Siripornadulsil 2013), resulting in decreased biomass, even though, the microbial populations here referred corresponds to rhizospheric community. The biomass of CdtB might decrease when higher concentrations of the metal are present in soil, compared to bulk soil. However, the size of the culturable bacterial community remains stable within rhizosphere populations covered with hyperaccumulator plants (Weyens *et al*. 2013; Lucisine *et al*. 2014). The size of culturable bacterial community is mostly counted on synthetic media containing chemical compounds of Cd in soluble (Schlegel *et al*. 1961; Mergeay 1995) and non‐soluble forms (Atlas 2010; Aleksandrov *et al*. 1967) founds in bulk soil, where nitrate is the least used form as sulphates are found more often (Bataillard *et al*. 2003; see Table 2). Therefore, the design of new synthetic media using chemical forms more aligned to the conditions found surrounding hyperaccumulator plants (such as rice or cacao) requires assessment.

### How active are CdtB in the rhizosphere?

The phylogenetically unrelated CdtB that form the Cd‐tolerant group are very active in polluted soils, when compared with other members of the total community. The group´s populations increase with increases in Cd concentration due to their tolerance, resilience capacities and prior stability (Griffiths and Philippot 2013; Xiao *et al*. 2020). In this way, both, the transcriptional factors involving physiological responses of tolerance and adaptation to the environmental disturbance of CdtB role are key elements to understand how active they are. Nonetheless, the replacement of sensitive microorganisms by a few resistant species can have serious ecological consequences for plant nutrition in farmlands, unbalancing essential elements uptake (i.e. Fe, Mn, Mg) by crops (van Beelen and Doelman 1997).

**Figure 2:**
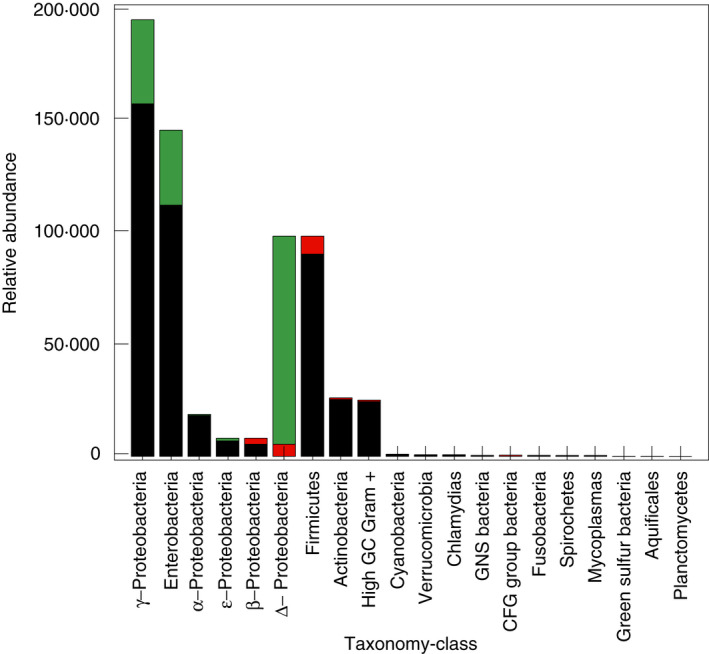
Relationship between operational taxonomic units (OTUs) and the class taxonomy of representative cadmium‐tolerant bacteria (CdtB) isolated from agricultural crops, taking into account two metabolic pathways (bioleaching and biotransformation) and the expression of three related genes *cadA*, *cadD* (Naz *et al*. 2005) and *zntA* (Beard *et al*. 1997) (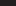 *cadA*; 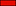 *cadD*; 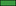 *zntA*).

Assessing the activity of CdtB, the study related (Xiao *et al*. 2020), shows evidence of enzymatic activity related to Cd metabolization (including urease, sucrase and acid phosphatase) and great impact on the growth of certain bacterial populations such as the actinomycetes in soil. The active populations, measured by assays of these enzymes (urease, sucrase and acid phosphatase), were identified as order bacteria >actinomycetes >fungi, where populations highly resistant to Cd amendments were comprised of Proteobacteria and Firmicutes, when exposed to a concentration gradient of 11·2, 16·8, 22·4 and 28 mg kg^−1^ of Cd^2+^ in soils samples during 4 days at 30°C (Xiao *et al*. 2020). The functional analysis of microbial communities was performed using the Biolog‐ECO microplate system and the functional diversity of the microbial community in soil was analysed using the Simpson index, Shannon diversity index and McIntosh index. Regarding the active populations, one of three enzyme activities assessed was strongly inhibited (urease with 94·3%) by the addition of 28 mg kg^−1^ Cd, indicating that urease activity might be a useful indicator of Cd soil pollution, as pointed out in recent studies within soil farmlands (Wang *et al*. 2019). Noteworthy, urease is not known to be inhibited by Cd ions (as many other enzymes are) (BRENDA 2021), thus, making the importance of this enzyme even more profound. At the same concentration, the CdtB biomass production increased only 3% from 14·2 × 10^7^ to 22·4 × 10^7^ colony‐forming units (CFU)·per ml. The urease activity as a bioindicator of heavy metals contamination has been described earlier (Wittekindt *et al*. 1996; van Beelen and Doelman 1997). Another study (Rogers and Li 1985) was even more related to the effect of Cd, measuring with the relationship to the loss of the soil dehydrogenase activity as percentage of control at several concentrations of Cd by the triphenyl tetrazolium chloride assay (TTC). The study shows a decrease from 100 to 3% of enzymatic activity when concentrations of Cd were added in a range between 0 to 3000 mg kg^−1^. Another study (Wu *et al*. 2019) shows that the use of a combination between biochar and PGPR bacteria (Plant Growth‐Promoting Rhizobacteria) will increase both microbial biomass and the activity of key enzyme activities measured by the fluorescein diacetate assay (FDA). This assay included acid phosphate, FDA hydrolase, invertase and urease to prevent Cd toxicity of vetiver grass (*Chrysopogon zizanioides* L.). To measure functional activity directly, the metabolic rate and thus the potential immobilization of Cd by tolerant or resistant populations could be carried out using the isothermal microcalorimetry (IMC) assay, which is well correlated with the TTC and FDA assays, as shown in recent studies (Bravo *et al*. 2018; Braissant *et al*. 2020). Therefore, the capacity of Cd metabolization will depend on the genes expression along key metabolic pathways, Cd concentration and the plasticity of CdtB to interact with the surroundings. Figure 2 shows the distribution of taxonomic‐classes of CdtB based on operational taxonomic units of three genes (*cadA*, *cadD* and *zntA*), related to two metabolic pathways (bioleaching and biotransformation) and two metabolic activities (Cd^2+^‐exporting ATPase and Cd‐translocating P‐type ATPase), as reported in references of Table 3.

**Table 3:** Distribution of most relevant reads of bacterial genes related to ‘relative abundance’ involved with cadmium tolerance showed by taxon groups. The new standardized bacterial taxonomy was used according to Parks *et al*. (2018)

Taxon group	Relative abundance			References
	*cadA*	*cadD*	*zntA*	
Actinobacteria	25 246	685	59	Nouioui *et al*. (2018)
Aquificales	38			Lin *et al*. (2019); Salam *et al*. (2020)
CFG group bacteria		3		Belimov *et al*. (2005); Zhang *et al*. (2008)
Chlamydiales (Verrucomicrobiota)	547	1	33	Luoma (1998)
Cyanobacteria	858	114	1	Jaakkola *et al*. (2016)
Enterobacterales	111 273	6	33 196	Palmer *et al*. (2010); Chen *et al*. (2016)
Firmicutes	89 592	7955	1423	Nucifora *et al*. (1989); Bashir *et al*. (2017); Gaultier *et al*. (2018)
Fusobacteriota	413	1	1	Chen, Zheng, Ding *et al*. (2017)
Chlorobia	39		1	Shi *et al*. (2019); Wang *et al*. (2019)
Chloroflexi	468			Minari *et al*. (2020)
Acidobacteriota	24 247	679	52	Khan *et al*. (2017); Parsons *et* *al*. (2020)
Mycoplasmatales	111	1		de Zwart *et al*. (1991); Oneal *et al*. (2008)
Planctomycetota			1	Lage *et al*. (2012); Chen, Zeng, Xu *et al*. (2017); Chen *et al*. (2019)
α‐Proteobacteria	18 081	58	447	Ormeño‐Orrillo *et al*. (2012)
γ‐Proteobacteria (formerly β‐proteobacteria)	5378	1175	17	Han *et al*. (2011)
γ‐Proteobacteria	156 086	55	37 253	Buell *et al*. (2003)
δ‐Proteobacteria		5	2	Naz *et al*. (2005)
ε‐Proteobacteria	6863		2	Cornelius *et al*. (2021)
Spirochaetota	127		1	Hardham and Rosey (2000)
Verrucomicrobiota	755			Luo *et al*. (2019)

### CdtB as Cd stress—controllers in farmlands

Regarding the maximum Cd concentrations in agricultural soils (Commission 2007; FAO/WHO 2018; Hou *et al*. 2019), the *Codex Alimentarius Commission* of the joint FAO/WHO Food Standards program proposed a maximum permissible concentration of Cd in polished rice and cocoa‐derived products (i.e. 0·4 or 0·8 mg kg^−1^ for rice and chocolate with % cacao solids, respectively). Therefore, the use of CdtB has now become relevant for the bioremediation of agricultural soils even at low Cd concentrations (Makino *et al*. 2019). The importance of CdtB lies in its capacity to interact with Cd in soils (microbiogeochemistry) to form nucleating interfaces. These, microbial interfaces are involved in a wide range of geochemical reactions (Beveridge 1989). In these reactions, the role of physicochemical properties of soils/sediments, such as soil organic matter (SOM), phosphorus, potassium content (Bravo *et al*. 2018) and Ca organo‐mineral association (Rowley *et al*. 2021) of farmlands is highlighted. The interfacial effect of CdtB increases the reactions that results in bioweathering of Cd. Bioweathering causes anhydrous and crystalline early‐stage forms of Cd minerals in bacterial surfaces (Beveridge 1989) and decreases the metal concentration from soil solution.

This is of particular relevance regarding the new regulations or laws imposing limits on the maximum allowable Cd content in soils. For example, the ‘agricultural land soil prevention law’ in Japan (Makino *et al*. 2019) states a standard of ≤0·4 mg kg^−1^ for the Cd concentration in rice grains, whereas the ‘soil contamination countermeasures law’ in the same country (Makino *et al*. 2019), stipulates 150 ± 0·5 mg kg^−1^ of Cd in rice field soils, a considerable difference from the regulation for grains. The soil contamination countermeasures law is also applicable to other agricultural soils in that country.

Multifaceted solutions, that include, the incorporation of CdtB, might be included in a strategy aimed at minimizing the accumulation of the labile fraction of Cd in soils into the grains due to the differences in thresholds in each matrix (soil or grains/beans, depending on the crop). Determination of the maximum reduction in soil Cd content by CdtB *in situ* is very difficult; hence, use of active CdtB is needed because of their capacity helping in addition with other amendments (i.e. dolomite or biochar application), to decrease the available Cd, that might enter plant tissues. This reduction occurs because bioavailable Cd in soils is affected by CdtB through seven major mechanisms (see Table 4). This will be further detailed in the next sections.

**Table 4:** Some examples of culturable and viable cadmium‐tolerant bacteria/yeast/algae showed by the tolerance mechanism and the associated crop/niche system where they were isolated

Tolerance mechanism	Type of micro‐organism	Strain	Crop system/niche	Reference
Biosorption	Algae	*Fucus vesiculosus*	Estuaries	Herrero *et al*. (2005)
	Algae	*Gelidium sesquipedale*	Industrial algal waste	Vilar *et al*. (2006)
	Algae	*Oedogonium hatei*	Stream	Gupta *et al*. (2010)
	Bacteria	*Aeromonas caviae*	Potable groundwater supplies	Aryal (2020)
		*Aeromonas amylolyticus*	Agricultural water	Aryal (2020)
		*Alishewanella* sp.	Paddy soils	Wang *et al*., (2020)
		*Bacillus cereus* RC‐1	Cadmium‐contaminated paddy soil	Aryal (2020)
		*Bacillus thuringiensis* OSM29	Industrial effluent contaminated soil	Aryal (2020)
		*Brevundimonas* sp. ZF12	Hot‐spring waters, potato	Aryal (2020)
		*Corynebacterium glutamicum*	Bearing solutions, lettuce	Aryal (2020)
		*Escherichia coli* P4	Porcine origin	Aryal (2020)
		*E. coli* HD701	Industrial waste, sugar cane	Aryal (2020)
		*Enterobacter* sp.	Industry wastewater treatment plant	Lu *et al*. (2006)
		*Geobacillus therantarcticus*	Groundwater, barley, wheat	Aryal (2020)
		*Ochrobactrum anthropi*	Activated sludge, paddy fields	Aryal (2020)
		*Pantoea* sp. TEM18	Wastewater treatment petrochemical industry, rice	Aryal (2020)
		*Pectobacterium* sp. ND2	Soil of the industrial zone, potatos	Aryal (2020)
		*Pseudomonas aeruginosa* B237	Zinc mine, rice grains	Aryal (2020)
		*Pseudomonas chengduensis* MBR	Farmland soil, paddy soils	Wang *et al*. (2020)
		*Pseudomonas fluorescens*	Cotton, wheat, tobacco	Yu *et al*. (2007)
		*Pseudomonas plecoglossicida*	Sludge, maize‐wheat cycles	Aryal (2020)
		*Pseudomonas stutzeri*	Activated sludge, soybean plants	Aryal (2020)
		*Staphylococcus xylosus*	Contaminated soil in a mining industry	Ziagova *et al*. (2007)
		*Streptomyces rimosus*	Zn enriched soils, sweet potato residue, pomelo peel	Aryal (2020); Selatnia *et al*. (2004); Saikaew *et al*. (2009)
	Cyanobacteria	*Spirulina* sp.	Lake, irrigation water	Solisio *et al*. (2008)
	Yeast	*Saccharomyces cerevisiae*	None	Chen and Wang (2007)
Bioleaching	Bacteria	*Acetobacter* spp.	Forest soils	Hou *et al*. (2020)
		*Acidithiobacillus* spp.	Forest soils	Hou *et al*. (2020)
		*Arthrobacter* spp.	Forest soils	Hou *et al*. (2020)
		*Burkholderia* sp. Z90	Industrial soil	Yang *et al*. (2018)
		*Cytobacillus oceanisediminis* 2691	None	Kim *et al*. (2016)
		*E. coli* DH5α	None	Kim *et al*. (2016)
		*Pseudomonas* spp.	Forest soils	Hou *et al*. (2020)
		Sulphur‐oxidizing bacteria (SOB)	Paddy soils irrigation	Hou *et al*. (2020)
	Cyanobacteria	Cyanobacteria	Agricultural water	Bolan *et al*. (2014)
Biotransformation	Bacteria	*Alishewanella* sp.	Paddy soils	Wang *et al*. (2020)
		*Brevundimonas* sp. KR013	Pastureland	Shukla *et al*. (2019)
		*Mesorhizobium huakuii* subsp. Rengei B3	*Astragalus sinicus*	Shukla *et al*. (2019)
		*Micrococcus* sp.	*Glycine max* L.	Yasmeen *et al*. (2019)
		*Pseudomonas chengduensis* MBR	Farmland soil	Wang *et al*. (2020)
		*Pseudomonas* sp. KR017	*Brassica juncea* (L.), irrigation water	Shukla *et al*. (2019); Singh *et al*. (2020)
		*Rhizobium leguminosarum* bv *trifolii* NZP561	*Trifolium repens* L.	Shukla *et al*. (2019)
		*Serratia* sp.	*Solanum igrum*	Yasmeen *et al*. (2019)
	Cyanobacteria	Cyanobacteria	Agricultural water	Bolan *et al*. (2014)
Biodegradation	Bacteria	*Acidithiobacillus ferrooxidans*	Paddy soils	Abbas *et al*. (2018); Xu *et al*. (2019)
		*Acidiphilium symbioticum*	Paddy soils	Abbas *et al*. (2018); Xu *et al*. (2021)
		*Actynomicetes*	Pastures	Xiao *et al*. (2020)
		*Arthobacter viscosus*	Agricultural wastewater	Abbas *et al*. (2018)
		*Bacillus laterosporus*	Wasterwater stream	Abbas *et al*. (2018)
		*Bacillus licheniformis*	*Spinacia oleracea* L.	Abbas *et al*. (2018); Asif *et al*. (2020)
		*Enterococcus faecium*	None	Abbas *et al*. (2018)
		*Staphylococcus aureus*	None	Abbas *et al*. (2018)
Bioweathering	Bacteria	*Bacillus subtilis*	Cowpea	Beveridge (1989); El‐Nahrawy *et al*. (2019)
		*Bacilus licheniformis*	*Spinacia oleracea* L.	Beveridge (1989); Asif *et al*. (2020)
		*Burkholderia* sp.	Cacao rhizosphere (*Theobroma cacao* L.)	Bravo *et al*. (2018)
		*Enterobacter* sp.	Cacao rhizosphere (*Theobroma cacao* L.)	Bravo *et al*. (2018)
		*Pseudomonas aeruginosa*	None	Beveridge (1989)
		*Acetobacter* spp.	None	Hou *et al*. (2020)
		*Acidithiobacillus* spp.	None	Hou *et al*. (2020)
		*Arthrobacter* spp.	None	Hou *et al*. (2020)
		*Pseudomonas* spp.	None	Hou *et al*. (2020)
		Sulphate‐reducing bacteria (SRB)	None	Hou *et al*. (2020)
Chemisorption	Bacteria	*Aquaspirillum* spp.	None	Beveridge (1989)
		*Bacillus cereus* RC‐1	Cadmium‐contaminated paddy soil	Aryal (2020)
		*Leptothrix* sp.	Lakes	Beveridge (1989)
		*Sphaerotilus* sp.	Waste stream	Beveridge (1989)
		*Sporosarcina ureae*	None	Beveridge (1989)
Bioaccumulation	Bacteria	*Aquaspirillum magnetotacticum*	Paddy soils	Beveridge (1989)
		*Burkholderia* sp.	Rose garden	Lee (2020)
		*Cupriavidus taiwanensis*	Rice (*Oryza sativa* L.)	Yasmeen *et al*. (2019)
		*Delftia tsuruhatensis*	Thai jasmine rice	Yasmeen *et al*. (2019)
		*Kluyvera ascorbata* SUD165 & SUD165/26	Tomato	Shukla *et al*. (2019)
		*Methylobacterium oryzae*	Tomato	Yasmeen *et al*. (2019)
		*Pseudomonas tolaasii* RP23	Canola	Shukla *et al*. (2019)
		*Pseudomonas fluorescens* RS9	Perennial grasses (Graminaceae)	Shukla *et al*. (2019)
		*Rhodobacter sphaeroides*	Wheat seedlings	Yasmeen *et al*. (2019)
		*Stenotrophomonas acidaminiphila*	Rice (*Oryza sativa* L.)	Yasmeen *et al*. (2019)
		*Variovorax paradoxus*	Indian mustard (*Brassica juncea* L. Czern.)	Shukla *et al*. (2019)
	Yeast	*Saccharomyces cerevisiae*	Wastewater	Lee (2020)

## Biochemistry and physiology of CdtB

**Figure 3:**
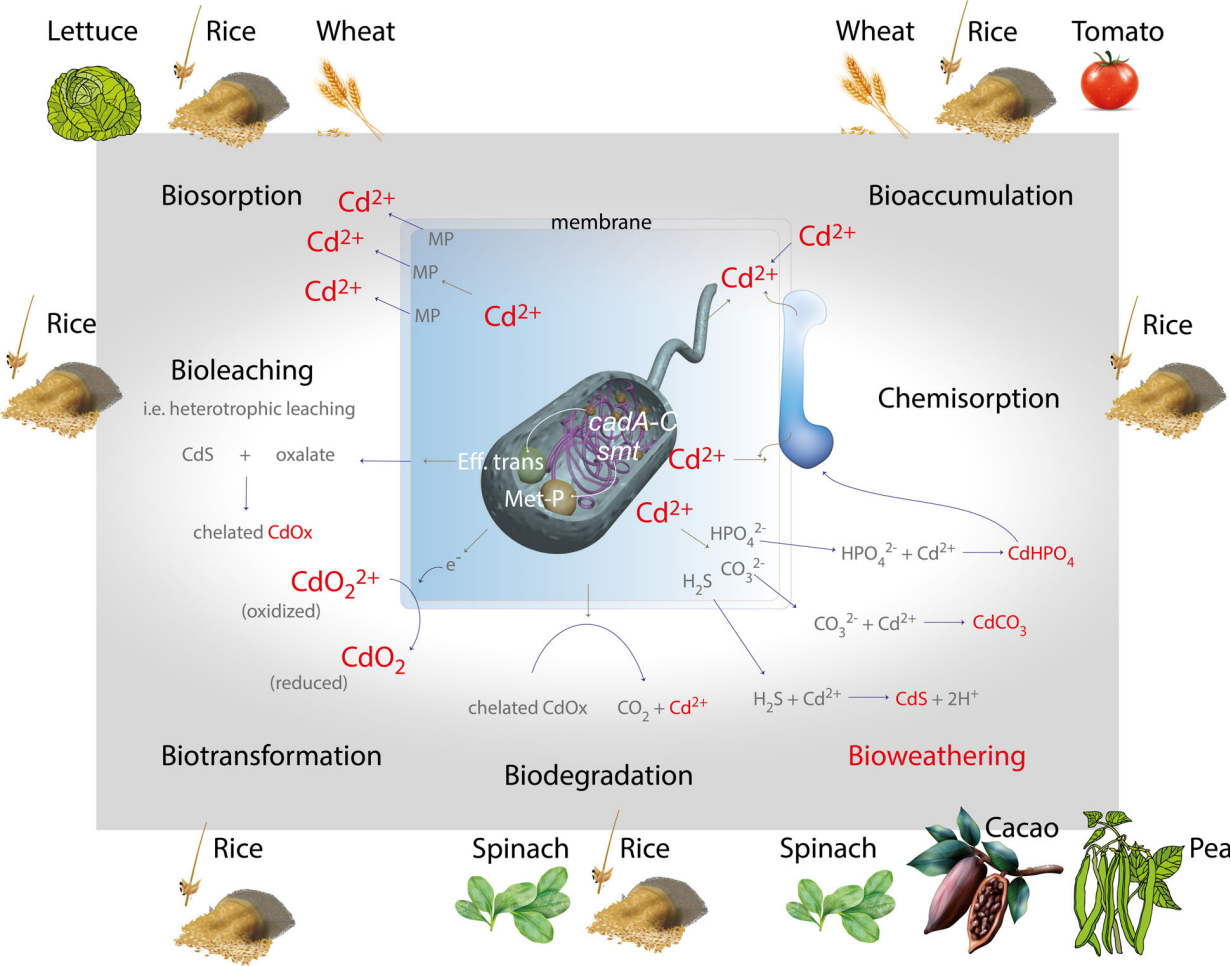
Sketch illustrating the biochemical mechanisms of CdtB active in crop‐growing soils. The biochemical paths yield geostable chemical species of Cd.

To the best of our knowledge, CdtB can exploit seven main mechanisms resulting in geostable chemical species of Cd contributing as Cd detoxification routes (Abbas *et al*. 2018). Table 4 shows some examples of Cd tolerance mechanisms, including a few examples of the most relevant bacterial strains attributed to each mechanism. Figure 3 shows an overview of Cd metabolic activity of CdtB in their surroundings.

### Biosorption

Biosorbents are biological materials able to accumulate heavy metals from environment. Biosorbents include the materials derived from different biological sources like agricultural by‐products a mixture of chitin, peat and microbial biomass, which are responsible for the acid–base interactions, chelation, complexation, electrostatic interactions, ion‐exchanges and van der Waals interactions, as the main soil processes (Singh *et al*. 2020). Moreover, these biomaterials interact with mineral adsorbents, such as clay and zeolites occurring in soils, to immobilize Cd. Biosorption can also be attributed to the microbial community, including CdtB populations. According to the literature, microbial biomass can bind heavy metals either actively, or passively or by a combination of both processes (Ansari and Malik 2007), depending on the biosorbent population used. The microbial biomass consisting of CdtB could enhance the biosorption capacity using its binding ability, i.e. the electrostatic attraction between Cd ions and negatively charged reaction sites on the bacterial cell wall or the exopolymers produced (EPS) will pave the way for the formation of biological bioadsorbents of Cd, such as chitin and chitosan‐like (Ayangbenro and Babalola 2017; Singh *et al*. 2020), or CdS nanoparticles (NPs) (Xu *et al*. 2021). For instance, a study has shown that strains *Microbacterium* sp. D2‐2 and *Bacillus* sp. C9‐3 exhibited a maximum biosorption capacities of 222·22 and 163·96 mg of Cd^2+^ per gram of biomass at pH 5, respectively (Long *et al*. 2021). The authors suggested that the hydroxyl, carboxyl, carbonyl and amino groups on both strain’s biomass were the main binding sites for Cd. An interesting amount of work has been done regarding Cd binding to bacterial cell wall in several CdtB strains, such as *Pseudomonas putida*, *Shewenella oneidensis*, *Rhizobium tropici* and *Agrobacterium* sp. (Kenney and Fein 2011b), which highlighted the role of extracellular exopolysaccharides (EPS) in Cd binding formation and metal absorption onto bacterial wall cells, with functional groups that include the same for biomass related above, plus the proton active sulfhydryl groups. The key role of these EPS production is Cd sequestration reducing the bioavailability of the metal. Interestingly, the percentage of Cd adsorbed by the CdtB above mentioned (*P. putida*, *S. oneidensis*, *R. tropici* and *Agrobacterium* sp.) increases in function of pH and rises to 100% at pH 9·7 in *P. putida*, 9·5 in *S. oneidensis*, 10·0 in *R. tropici* and 11·0 in *Agrobacterium* sp., whereas the *P. putida* EPS rise the 8 × 10^5^ mol l^−1^ of Cd adsorbed at pH 8·5. Another study (Kenney and Fein 2011a) was conducted performing potentiometric titrations and Cd adsorption experiments using two acidophilic CdtB (*Acidiphilium cryptum* and *Acidiphilium acidophilum*) and two alkaliphilic CdtB (*Bacillus pseudofirmus* and *Bacillus circulans*) to determine their Cd‐binding capacities. The study concludes that no matter the extreme or neutral environment the bacteria have, similar proton and metal adsorption behaviour could be found, stating that a single set of proton and metal binding constants can be used to model the behaviour of bacterial adsorption in a wide range of natural environments, which includes agricultural soils. In this context, the isothermal titration calorimetry studies have also shown a remarkable Cd absorption capacity of CdtB (Qu *et al*. 2021), where, the molecular binding mechanisms and distribution of Cd onto goethite, humid acid, *P. putida* cells and their composites at different mass ratios were studied. The above‐mentioned study demonstrates an enhanced Cd adsorption of between 10 and 30% of the metal when Cd was added at 5 mg l^−1^, where more than 93·5% of the adsorbed Cd was bound onto the goethite‐humid acid fraction.

### Bioleaching

Bioleaching is one of the most widely used strategies in bioremediation, because is an environmentally friendly and cost‐effective technique for remediation of severely Cd‐contaminated farmlands. Soil leaching is an attractive method for soil remediation due to its simplicity of operation, low cost and high efficiency (Yang *et al*. 2018). Bioleaching includes the transformation of insoluble Cd added to an organic acid into a soluble metal chelate (Singh *et al*. 2020). In this way, indigenous CdtB are able to remove Cd from their adjacent soil microenvironment. Bioleaching, thus, removes Cd from soils by chelating the metal with functional groups and transforming Cd speciation fractions to increase their mobility (Gadd 2010). Bioleaching could be addressed by EPS produced by bacterial wall cells, which are polysaccharides, lipids, lipopeptides, glycolipids and neutral lipids, exhibiting pronounced surface activity to interact with heavy metals such as Cd (Yang *et al*. 2018). As shown in the study of Yang *et al*., assessing a *Burkholderia* sp. strain Z‐90 with contaminated mining soils, the main mechanism of bioleaching remains the complexation of Cd with carboxyl groups, depending on the pH of the leaching solution (around 5·0) and the leaching time (around 5 days). Nonetheless, the bioleaching mechanism requires a further step, such as the flocculation of Cd with polyaluminium chloride, since Cd has been demonstrated to be highly available after bioleaching remaining in the acid‐soluble fraction of contaminated soils.

Another important CdtB microbial group that participates in the bioleaching of Cd is that of the sulphur‐oxidizing bacteria (SOB). The SOB‐CdtB bioleach Cd by increasing the pH, producing more chelators and allowing the sulphate to react with Cd through sulphur oxidation, to form insoluble minerals of Cd, which have been also isolated from metallurgical industry wastewater (Wu *et al*. 2020).

Interestingly, in agricultural soils, SOB species have been found associated to bioleaching, such as *Acidithiobacillus* spp., *Acetobacter* spp., *Arthrobacter* spp. and *Pseudomonas* spp. Fungi species, such as *Penicillium* spp., *Aspergillus* spp. and *Fusarium* spp, also have been highlighted to bioleach Cd (Hou *et al*. 2020).

Although *Burkholderia* sp. Z‐90 has been proposed for biotechnological scale‐up processes, for soils with high Cd content for mine site remediation, at present, no *Burkholderia* spp.*, Enterobacter* spp. or *Pseudomonas aeruginosa* have yet been used in agricultural soils. Indeed, pathogens are unlikely to be used for agricultural purposes because of the potential risks to humans or harvest. Interestingly, microorganisms such as *Burkholderia* do exhibit great Cd immobilization ratios and a genetically modified organisms (GMOs) including *Burhkolderia* spp. could become to be of interest for future suitable applications. For instance, in certain crops, it is possible that wild‐type bioleaching population might have a low remediation ability or a lower tolerance to high metal concentrations. In such context, the use of GMOs with high capability to perform bioremediation of Cd by adding functional genes related to immobilization, could be an interesting option to consider when applied under strictly controlled conditions. Still, high horizontal gene transfer might be an issue when the GMOs are exposed to autochthonous microbial populations (Bhayani *et al*. 2020). Furthermore, the use of GMOs is not well regulated and deserves more attention due to potential human health issues. The bottleneck of using GMOs in agricultural heavy metal remediation will be addressed in the last section of this review.

### Biotransformation

The transformation of Cd ions on the surfaces of biological cells is the first step in the interaction between the metal and the bacterial cell (Wang *et al*. 2020). Cd ions undergo various biochemical reactions, migration and transformation processes at the cell–metal interface. One of these involves the production of EPS, which might be a key feature of Cd tolerance (Barken *et al*. 2008). In particular, uronic acid‐rich EPSs have high Cd‐binding ability (Abbas *et al*. 2018). Some studies have described redox changes of Cd in some SRB that produce biofilms to protect themselves against Cd ion stress. These organisms transform Cd into nanosized serpentinite microsphere aggregates (Covelo *et al*. 2007), whereas sphalerite (ZnS) NP aggregates (Labrenz *et al*. 2000). In this study, the microbiological, geochemical and mineralogical interactions leading to ZnS biomineralization in complex natural systems were assessed within a biofilm community (*in situ*) that include the sulphate‐reducing species *Beggiatoa* sp., *Desulfobacter* spp. and *Desulfobacterium* spp. collected from a flooded tunnel within carbonate rocks. SRB are highlighted in the biotransformation mechanism, because it transforms sulphate into sulphide, then the sulphide produced reacts with the surrounding available Cd to produce CdS found as greenockite or hawleyite forms (Castillo *et al*. 2012b; Liu *et al*. 2020), or even as an impurity substituent of sphalerite or wurtzite (Castillo *et al*. 2012a), as a later insoluble product.

### Biodegradation

Cadmium‐tolerant bacteria are also known to have a metabolic function in hydrocarbon biodegradation (i.e. polycyclic aromatic hydrocarbons or PAHs), mediated by Cd inhibition in contaminated experiments (Hoffman *et al*. 2005; Thavamani *et al*. 2015). Another study (Shi *et al*. 2013), demonstrated that even though higher concentrations of Cd results in a decrease in the growth of a strain of *P. aeruginosa*, the biodegradation rate of deca‐bromo‐diphenyl ether (BDE‐209) at 1 mg kg^−1^ of Cd is greater than at other Cd contents assessed. In this study, most of the added Cd was complexed as CdHPO_4_, which is generally reported to be non‐bioavailable. This confirms the role of CdtB in the biodegradation of PAHs from contaminated sites.

In farmlands such as the cacao plantations nearby coal mines and hydrocarbon pipelines observed in cacao‐producing countries, such as Colombia, and Ecuador, the anthropogenic contamination due to the released PAHs is frequently observed (Barraza *et al*. 2017; Bravo and Benavides‐Erazo 2020) with high P soil content. Thus, the use of this particular group of CdtB with PAHs degradation capacity in the presence of elevated concentrations of Cd in farmlands (greater than 1 mg kg^−1^) could be used. Microbial tolerance to Cd may be related to the occurrence of metallothionein proteins, which can bind Cd and Zn ions (Jjemba 2004; Shukla *et al*. 2019). Thus, Cd tolerance in microbial cells would be mainly acquired through specific active efflux pumps via an energy‐dependent mechanism to pump out Cd cations (Jjemba 2004; Shukla *et al*. 2019), using metallothioneins with the capacity to bind Zn^2+^/Cd^2+^ due to their cysteine enrichment (Abbas *et al*. 2018).

### Bioweathering

The two biogeochemical mechanisms biomineralization and bioweathering could influence Cd precipitation or availability in the subsurface soil solution in farmlands, including cacao farms (Bravo *et al*. 2018). Bioweathering has been defined as the dissolution of rocks and mineral substrates carried out mainly by microorganisms (Gadd 2007; Mapelli *et al*. 2012). The bioweathering process is a continuous formation and deposition of mineral insoluble forms of the sequestered Cd. This is mediated by the metabolic activity of microorganisms inducing a change of the environment (Dove *et al*. 2018), in the subsurface layer. This could decrease the Cd assimilation ratio in cacao roots, as well as accumulation in the cocoa beans (Bravo *et al*. 2018). In this case, bioweathering implies the conversion of a soluble source of Cd, such as CdCl_2_ or CdNO_3_, into a less or non‐soluble form, such as CdO, Cd‐β(OH)_2_, CdCO_3_, CdSO_4_ or CdSe, which are secondary forms arising from bacterial metabolic activity, as supported by evidence found in cacao‐growing farms (Bravo and Benavides‐Erazo 2020). A similar reaction occurs with CaCO_3_ derived from the bacterial conversion of calcium oxalate (CaO_x_), and this also makes an important contribution to biological mineralization (Braissant *et al*. 2004; Bravo *et al*. 2011). Interestingly, the chemical speciation changes mediated by CdtB influence the water solubility of the end products. This is an important criterion of remediation because once the metal is precipitated into crop‐growing soils, its biotranslocation and bioaccumulation rates in plant tissues are also reduced. Therefore, the end products of this biogeochemical pathway yield geostable Cd chemical species that stay in the ground.

### Chemisorption

This mechanism is also termed biosorption because it refers to the adhesion of solutes (mainly Cd ions) onto molecules of biological origin (mainly proteins or peptides) that occur at the biomass surface. For a more in depth understanding of bio‐ and chemisorption processes, the reader is referred to a recent review (Aryal 2020). In crop‐growing soils, the ratio of Cd / Fe, / Mn or /organic matter content has an important influence on metal chemisorption. Bacterial chemisorption occurs when the elemental composition in soils favours a Cd gradient that will adsorb due to the metabolic activation of the Cd effluent system. Interestingly, the same system is used for zinc stress reduction. Hence, bacterial tolerance of Cd is accomplished via two efflux mechanisms, (i) using the P‐type ATPase pump system (see ‘Eff trans’ in Figure 3) and (ii) using a resistance‐nodulation‐cell division protein family (RND)‐driven transporter mechanism, which is involved in the transit of metals such as Cd (Shukla *et al*. 2019). The P‐type ATPase effluence system causes transport of the metal by ATP hydrolysis, so the reaction is considered endothermic, due to the need for energy to hydrolyse ATP. By comparison, the RND‐driven transporter does not derive energy through ATP hydrolysis to convey the metal into the bacterial cell. It is worth to mentioning that fluctuations in energy availability can alter microbial activity related to Cd (Hart and Gorman‐Lewis 2021). This highlights the expression of the *smt* and *zntA* genes that are related to metabolic pathways unrelated to ATPase systems, which could be expressed by the activation of the *cad* operon. Moreover, in bioprecipitation the plasmide‐borne *czc* operon encoding for a chemiosmotic proton antiporter‐mediated efflux of cations, plays an important role in ensuring Cd tolerance (among others, such as Co, Zn), through a tricomponent export pathway studied in the facultative chemolithotroph bacterial strain *Alcaligenes eutrophus* CH34 (Diels *et al*. 1995). This metabolic pathway is activated in the presence of Cd and as result, the removal of Cd from culture supernatant fluids through precipitation of carbonates (CdCO_3_) and hydroxides (see Table 1) was observed (Diels *et al*. 1989, 1995).

### Bioaccumulation

In this mechanism, the membrane transport system related to manganese is important, as highlighted previously (Smiejan *et al*. 2003). The bioaccumulation Cd uptake models, including the free ion activity model and the biotic ligand model, assume that biological internalization is rate limiting and first order and that toxicity can be related to bacterial cell wall uptake fluxes (Smiejan *et al*. 2003). The transport rate across the membrane (uptake flux) can be assumed directly proportional to the free metal ion concentration in solution or to the concentration of surface transporter‐bound Cd. A recent study has shown that the bacterial strain *Burkholderia* sp. soil CdR15 is a wild‐type bacterium grown in uncontaminated soils which was artificially adapted to increasing Cd concentrations of up to 274·98 mg l^−1^ and is highlighted due to its bioaccumulation adaptative mechanism (Lee 2020).

Interestingly, some mechanisms described are also involved with fertilizing properties, such as K or P solubilization from insoluble mineral forms in agricultural soils (Sattar *et al*. 2019) using kaolinites, montmorillonites or micas just to cite some major sources in agricultural soils in tropical zones (Hou *et al*. 2020). The group of micas, including kaolinites and montmorillonites has shown removal capacity of Cd alone, up to 50 mg l^−1^ Cd (Gupta and Bhattacharyya 2006), or in combination with CdtB such as *Agrobacterium tumefaciens* (up to 100 mg kg^−1^ Cd) or *Bacillus megaterium*, up to 10 mg kg^−1^ Cd (Babich and Stotzky 1977). This is because these minerals have the capacity to accumulate several cations with similar cation interchange capacity (11·3 meq per 100 g for kaolinite and 153·0 meq per 100 g for montmorillonite).

## Biotechnological applications of CdtB in agricultural soils

Because the polluted farmlands are increasing worldwide, the development of cost‐effective technologies is needed. These might include bioremediation strategies, in combination with other soil dressing countermeasures, to tackle Cd issues for several crops, thereby improving the safety of the food chain. The idea behind the use of CdtB as a strategy for decreasing Cd content in soils comes from the basic fact that CdtB are represented by the diverse microbial populations that uniquely acclimated to concrete conditions of Cd presence in its environment, evolving either in one or more metabolic mechanisms to survive against toxicity. The best described operon by far is the *cad*, which, as mentioned previously, is related to the Type P ATP‐dependent protein of the bacterial wall, followed by the *met* genes, related to the production of the MET proteins found in the cytosol of CdtB (Aryal 2020).

CdtB should be applied in three major scenarios of Cd contamination: (i) in agricultural soils close to mining sites; (ii) in agricultural soils where atmospheric depositions occur due to coal or precious mineral extractions near the crop; and (iii) in agricultural soils with high inputs of organic matter where the use of manure or Cd‐containing chemical fertilizers (mainly P‐like and N‐like and not regulated) is common and there is no control of Cd content.

Depending on the scenario and physico‐chemical conditions of the soil and crop, specific groups of CdtB should be applied. For instance, for the first two scenarios, soil CdtB should be applied in combination with the mineral amendments, such as in rice field experiments (Shi *et al*. 2019), using plant growth‐promoting CdtB ‘PGP‐CdtB’ (Pishchik *et al*. 2002). This might induce a precipitation of insoluble geostable forms of the metal i.e. minerals such as Greenockite or Otavite (Bravo *et al*. 2018), or organic CdS chelates (Wiggenhauser *et al*. 2021). This is an important strategy for incorporating Cd ions into carbonates, phosphorites and sulphides, resulting in a Cd sink through bioweathering of associated rocky types. That is how the immobilization potential of CdtB increases the binding adsorption with the surrounding minerals, at the microscale to compete against the mobilization potential ratio of Cd (Kubier *et al*. 2019) as the most mobile heavy metal occurring in environments, such as the farmlands. In the third scenario, a combination of endophytic cadmium‐tolerant bacteria (ECdtB) from autochthonous populations from crop systems, such as it has been already tested in rice (Siripornadulsil and Siripornadulsil 2013; Cheng *et al*. 2021) some others in tomato (Madhaiyan *et al*. 2007), or in *Mimosa pudica* trees (Chen *et al*. 2008), or even more recently, in cacao trees (Bravo *et al*., In prep.), either with edaphic or foliar applications, will replace the amendment of contaminated phosphoric fertilizers of chemical‐based composition, especially in acidic tropical soils where high amounts of fluorapatites have been detected.

## The potential for bioremediation using CdtB in Cd‐contaminated agricultural soils

**Figure 4:**
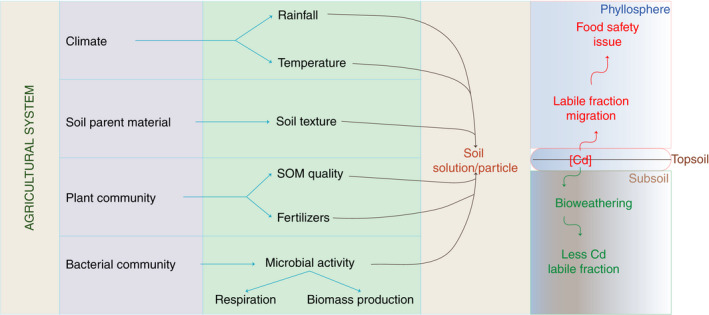
Sketch illustrating several activities involved in the use of cadmium‐tolerant bacteria (CdtB) in both agricultural and food‐safety strategies.

The primary source of Cd in unpolluted soils is the parent material (Mislin and Ravera 1986), mainly cretaceous sedimentary rocks and shales, which are frequent rock types found in acidic tropical farmlands (Traina 1999). However, the contribution of geogenic sources to Cd release in crops is probably low, at less than 0·3 mg kg^−1^ of Cd^2+^ (McGrath 1999), compared to other contributions due to human activities, that exceed the 0·10 mg kg^−1^ maximal concentrations suggested overall for agricultural soil (Hou *et al*. 2019), or the 0·4 and 0·8 mg kg^−1^ Cd concentrations as shown for rice and derivatives of cocoa crops (Makino *et al*. 2019). At present, an increasing number of studies have focused efforts on implementing microbial‐based bioremediation strategies for several crops (Siripornadulsil and Siripornadulsil 2013; Bravo *et al*. 2018; Jan *et al*. 2019). However, the use of CdtB is clearly just one step toward mitigating the effects of Cd fluxes from soils into the food chain. Figure 4 shows some factors that could drive the use of CdtB, either for diagnostic purposes or for application at different stages of the value chain for food safety.

Nonetheless, despite the efforts taken by the international community regarding CdtB‐based bioremediation of farmlands, the uptake of this strategy is still lower than expected (Figure S1 shows the dynamics of papers written during the past 10 years on the topic of CdtB, their use in agriculture and the low production of peer‐reviewed scientific literature per crop, respectively). Hereafter, the following are just a few examples.

### Cacao crops

In some developing countries, cacao culture is one of the largest economic activities and has an impact on social and political affairs. The *Codex Alimentarius* regulation (Commission 2007; FAO/WHO 2018) imposed the maximal permissible Cd content in chocolate and derivate products. Between some strategies to decrease the Cd content in cacao beans from certain Cd‐enriched areas in both Central and South America, that include the amendment of biochar or dolomite (Ramtahal *et al*. 2019; Bravo *et al*. 2021), the use of CdtB for bioremediation of Cd in Colombia is recognized as an important opportunity (Bravo *et al*. 2021; Bravo 2021); and other countries, such as Ecuador and Costa Rica, are just starting to develop experiments for bioprospecting of CdtB and for integrating further bioremediation setups within the national research agenda of public agricultural research institutions. The extensive microbial immobilization activity that CdtB strains, such as *Enterobacter* sp., have in cacao agroecosystems yield an immobilization removal up to 30% of Cd, when analysed in both laboratory and pot experiments (Bravo *et al*. 2018). These studies have shown the capacity of CdtB isolated from cacao‐growing farms assessed with up to 24 mg kg^−1^ Cd in the laboratory and 12 mg kg^−1^ assessed in the presence of cacao seedlings and autochthonous soils from the same farms where the CdtB were isolated. The formulation of a bioproduct based on CdtB and field applications of that bioproduct in cacao‐growing soils near cacao tree trunks is tuned on. Emerging needs for studies on heavy metals other than Cd, such as Pb levels in cacao beans and Pb effects on chocolate production (Ferreira de Oliveira *et al*. 2021) will require the use of Pb‐tolerant bacteria into postharvest of cacao.

### Lettuce crops

Some studies have pointed out the importance of CdtB to assess both Zn and Cd contents in lettuce growing soils (Rajapaksha and Amarakoon 2011). This work has assessed both bacterial and fungal populations with doses of up to 3 mg kg^−1^ Cd. In lettuce‐growing soils in Colombia, Rhizobiales strains, such as *Ensifer* and *Sphingobium*, are the main representatives of α‐Proteobacteria—culturable CdtB—whereas *Pseudomonas* spp. strains, belonging to the γ‐Proteobacteria, have exhibit interesting removal rates of Cd in media cultures (author’s personal observations). Interestingly, other authors have also found this CdtB useful for assessing Cd in lettuce crops (Xu *et al*. 2019). However, although enteropathogenic strains characterized such as *Serratia*, *Enterobacter* and *Raoultella* species exhibited higher Cd immobilization rates than the Rhizobiales group, these CdtB are not useful for bioproducts development.

### Pea crops

A recent study has shown the enhanced Cd tolerance in pea plants through the use of the CdtB *Enterobacter* sp. MN17 inoculated together with biochar and gravel sand (Naveed *et al*. 2020). The combined use of biochar and gravel sand with the bacterial inoculum resulted in an increase in plant height, shoot dry weight, root dry weight and seed weight. Likewise, protein, fat, fibre and ash were significantly increased with the combined use of CdtB and biochar/gravel sand. Therefore, in this particular crop, CdtB not only reduce the toxic effect of the metal, but it also increases nutritional factors for plant uptake.

### Rice crops

During the past 10 years, increasing studies have shown the potential of using CdtB as biofertilizer for Cd remediation in rice (Jin *et al*. 2021). Other studies have proposed the use of photosynthetic bacteria, such as *Rhodopseudomonas palustris* for Cd removal in paddy soil rhizosphere (Xiao *et al*. 2019). In that study, *R. palustris* immobilization of Cd not only inhibited its uptake by plants but also increased fresh weight of rice seedlings with 40 mg kg^−1^ of Cd in soil, as well as reducing the Cd uptake and subsequent accumulation in rice grains (from 0·65 to 0·19 mg Cd per kg of grains). Rice is perhaps the best studied crop in terms of the effects of heavy metals, and it has been used as a model crop for several reasons. One reason is that most of the human population bases its diets on rice or by‐products derived from this crop. Thus, there is interest in the use of CdtB, mainly sulphate‐reducing bacteria (Siripornadulsil and Siripornadulsil 2013). Other studies have shown that *Bacillus amyloliquefaciens* confers tolerance to various abiotic stresses, including Cd, by modulating rice plant response to phytohormones through osmoprotection and gene expression regulators (Tiwari *et al*. 2017). The sulphate‐reducing bacteria EPS production should be the key regulatory factor during Cd biotransformation. The CdtB *Cupriavidus taiwanensis* has become the most studied strain for bioremediation of rice crops (Siripornadulsil *et al*. 2014; Punjee *et al*. 2018).

### Wheat crops

PGPR CdtB have interesting abilities to immobilize Cd in wheat through ACC‐deaminase activity in Cd‐polluted growing soils (Zafar‐ul‐Hye *et al*. 2018). In that previous study, two concentrations of Cd were assessed (2·5 and 5·0 mg l^−1^) for the CdtB *Agrobacterium fabrum* and *Stenotrophomonas maltophilia*. The inoculation and concomitant ACC deaminase activity also showed an effect in wheat pigmentation.

Another point to note is that, in crop systems, several stages could be used to assess different populations of CdtB. For instance, while the main research was focused on soil CdtB, endophytic CdtB populations also exist that might have an impact in the phyllosphere or in seeds, grains or beans (the term depends on the crop system) that enter the food chain. Post‐harvest processes, such as fermentation of beans, roasting, deshelling and refining, are final steps in some crops during food transformation, and very little research into the use of CdtB is going on at these stages.

As indicated, several genera of CdtB could be used, depending on the crop system, the soil type, and the edaphoclimatic and ecological functions of the assessed system. Bacterial strains, such as *Enterobacter*, *Burkholderia* and *Agrobacterium*, have been identified as having substantial Cd tolerance. However, these genera are also related to plant diseases, which would affect their use when scaling up bioproduct manufacturing. Hence, a practical bottleneck has been here overseen.

### Promising approaches and trends to overcome key bottlenecks in Cd bioremediation

As mentioned, one of the major bottlenecks to resolving Cd presence in agriculture and the food chain is the use of ‘enteropathogenic bacteria’ or related phytopathogens. Although, it is possible to use allochthonous PGPB non‐pathogenic CdtB strains isolated from Cd hyperaccumulator plants colonizing metal contamined mining sites in semi‐arid soils, (i.e. the use of *Methylobacterium* sp. cp3‐mCherry, isolated from *Crotalaria pumila* seeds, which has Cd resistance genes, including the *czc* operon and Cd efflux proteins (Sánchez‐López *et al*. 2018), the adaptative and competitive challenges these populations have, in a non‐contaminated environment might decrease its Cd removal capacity in high specialized niches such as the agriultural soils (Montalbán *et al*. 2017; Bravo *et al*. 2018; Zhang *et al*. 2019). Interestingly, the most specialized CdtB reported from farms, with high ratios of Cd immobilization, seem to be species close to the enteropathogenic populations, i.e. in *Helianthus tuberosus*, with *Serratia* sp. 246 (Montalbán *et al*. 2017), or in paddy soils nearby manufacturing plants with *Burkholderia cepacia* GYP1 (Zhang *et al*. 2019), or in cacao soil plantations with *Enterobacter* sp. CdtB41 (Bravo *et al*. 2018). This is because genes encoding antibiotic resistance are related to operons implicated in biosynthetic pathways that deal with heavy metals, especially both the *cad* and *czc* operons related to Cd, copper and zinc regulation in both the periplasmic and cytosolic supra structure spaces. However, even if these types of CdtB are not useful in the development of bioproducts, this group could be approached from another angle.

Bioindicators or biosensors are another interesting field to be developed in the forthcoming years to produce fast diagnostic tools to detect Cd contamination in farmlands. One example is a genetically modified microbial biosensor constructed by inserting the *CadC* gene from *Bacillus oceanisediminis* 2691 into an *Escherichia coli* DH5α strain (Kim *et al*. 2016). This type of experiment is of particular interest for developing countries where the imposition of EU or FAO/WHO regulations requires several solutions regarding the issue of Cd mobility from farmlands into the food chains. Some bacterial genera are also not amenable to scaling up for bioproduction due to their phylogenetic relation to enteropathogenic species, i.e. *Enterobacter* species (Xu *et al*. 2017). However, due to its incredible metabolic ratio of tolerance to Cd and its relationship to antibiotic resistance (Zhao *et al*. 2020), this capacity might be used to build biosensors for early detection of Cd contents in several crop systems. Even if there are several methods to determine Cd in soils, or in other matrices such as plant tissues or fruits/grains, the use of biosensors is important in Cd detection, because this type of devices allow the minimization of sample and reagent volumes, rapid high‐resolution and high‐throughput analyses, high reproducibility and high portability (Kim *et al*. 2016). In that sense, the bottleneck for use of enteropathogenic populations disappears, because the genes of high tolerance, such as the *CadC* transcriptional regulators, could be inserted in non‐pathogenic high biomass yielding bacteria to analyse more soil samples in an efficient way, with low cost compared to spectrometric analyses, nonetheless, maintaining the accuracy of the Cd measurement, i.e., from 1124·11 to 5620·55 mg l^−1^ of Cd concentration (see (Kim *et al*. 2016), related to the levels of Cd may found in agricultural soils.

Another trend is the use of sulphate‐reducing bacteria, as these are responsible for modifying biochar through atomic interactions with available Cd in contaminated soils (Wu *et al*. 2019). In this case, sulphur‐iron‐rich niches in contaminated soils allow the assembly of the structure of the SRB community and its interaction either with biochar or other organic sources to form Cd micronodules of cadmium sulphide (CdS). Interestingly, in line with the formation of CdS, an electrochemical immunobiosensor was recently developed for ultrasensitive detections of quantum cadmium sulphides (CdS QDs) using GMOs, such as *E. coli* O157:H7 (Zhong *et al*. 2019). Likewise, regarding other applications, the bactericidal activity of metal oxide NPs, of microbial origin has been shown and is used in bioengineering and biomedicines. This could represent another window of use of CdtB against several forms of lethal microbes, e.g., using cadmium oxide NPs—CdO‐NPs (Azam *et al*. 2020)—or silver nanoparticles AgNPs, in postharvest applications on fruits/grains/beans. However, the removal of the NPs as well as the impact of NPs in human health, might be another bottleneck considering the silver is also toxic. Thus, more research should be developed in this field during the next decades.

In other cases, the use of inorganic sources, such as graphene oxide, has increase the adsorption of available Cd in soils. Nonetheless, increases in some populations of Acidobacteria and Actinobacteria can have a deleterious effect over critical functional groups of soil bacteria, such as nitrogen fixing and phosphorus or potassium solubilizing populations (Xiong *et al*. 2018). In another study, the addition of several minerals has also driven a change in soil bacterial community composition (Sun *et al*. 2016), increasing Actinobacteria populations. Despite *Actinomycetes* being related to improving the soil quality, the impact on other regulator populations in a resilient niche could have major effects ahead, including, in soil pH and cation exchange capacity fluxes (Mayanna *et al*. 2015). Therefore, an impact study should be conducted when inorganic amendments are applied with CdtB to determine their effect, once formulated, on nutrient cycling through the agroecosystem.

## Conclusions

As mentioned in the introduction, mobilization or immobilization of Cd may occur simultaneously in nature. The choice of a bioremediation strategy depends on a risk management assessment of the agroecosystem to be selected. Mobilizing activity can be used to enhance the removal of Cd through plant uptake and soil washing, whereas immobilizing Cd will reduce the transfer of this heavy metal into the food chain from plant uptake and reduce leaching into groundwater. It also depends on climatic factors, the farm altitude, the age of the crop, the genetics of hyperaccumulator plants, the farmer management and the proximity to mines or mineral extraction zones (Barraza *et al*. 2017; Bravo *et al*. 2021; Gil *et al*. 2021).

The increasing number of examples and approaches using CdtB as a mitigation strategy in farmland soils with elevated Cd concentration indicate that the natural methods will continue to be the most cost‐efficient and eco‐friendly way to remove Cd (Jacob *et al*. 2018), by activating critical biogeochemical pathways in the agroecosystem, such as the carbon cycle, due to microbial activity. What is trending now, is the integration of both organic/inorganic amendments that take advantage of either bioaugmented allochthonous populations or autochthonous CdtB, depending on the type of soils (i.e. acidic tropical soils where manganese oxides are highly desirable), and the adjusted cultural practices of farmers growing crops such as cacao, lettuce, pea, rice or wheat.

## Conflict of Interest

The authors declare no conflict of interests in the publication of this manuscript.

## Supporting information


**Figure S1**. Number of publications and year of publication of the past 10 years about CdtB in several crops. The keywords used for searching the target papers were: ‘cadmium’, ‘tolerant’, ‘bacteria’, ‘resistant’, ‘immobilization’. The software used was Vantage Point 2020 Search technology Inc, USA (Porter & Cunningham, 2004), VOSViewer (van Eck & Waltman, 2010), and the database were ISI WoS, Scopus, and Google Scholar. A private license of all software was used from the Corporación Colombiana de Investigación Agropecuaria AGROSAVIA though grant 1000664.
