# Supplementary figures and images for: Cadmium‐tolerant bacteria: current trends and applications in agriculture
Source: Lett Appl Microbiol. 2021 Nov 13;74(3):311–33. doi: 10.1111/lam.13594 (PMC9299123; doi:10.1111/lam.13594)

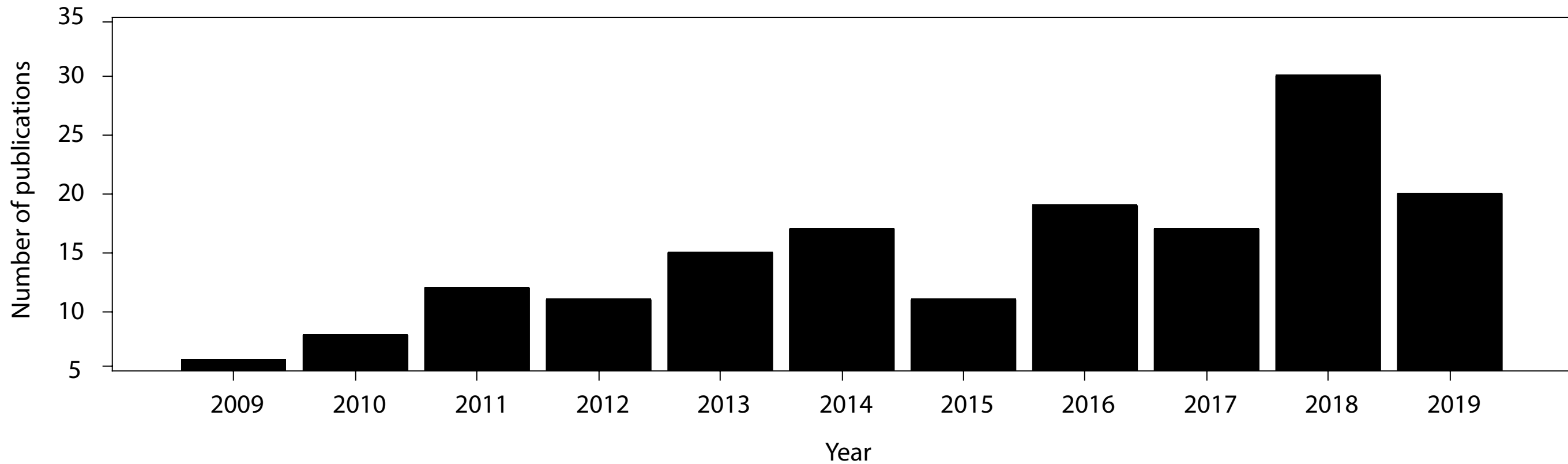

Supplement: Supplementary file 1 — Figure S1. Number of publications and year of publication of the past 10 years about CdtB in several crops. The keywords used for searching the target papers were: ‘cadmium’, ‘tolerant’, ‘bacteria’, ‘resistant’, ‘immobilization’. The software used was Vantage Point 2020 Search technology Inc, USA (Porter & Cunningham, 2004), VOSViewer (van Eck & Waltman, 2010), and the database were ISI WoS, Scopus, and Google Scholar. A private license of all software was used from the Corporación Colombiana de Investigación Agropecuaria AGROSAVIA though grant 1000664. [file LAM-74-311-s001.pdf]
